# Visualising the heart of chaos

**DOI:** 10.1038/s41377-021-00656-w

**Published:** 2021-10-14

**Authors:** Nicholas J. Lambert, Harald G. L. Schwefel

**Affiliations:** 1 Department of Physics, University of Otago, Dunedin, New Zealand; 2 The Dodd-Walls Centre for Photonic and Quantum Technologies, Dunedin, New Zealand

**Keywords:** Microresonators, Scanning probe microscopy

## Abstract

The intra-cavity electro-magnetic field distribution in a microdisk resonator can be visualised by inducing a phase shift via a scanning probe beam.

The intricate patterns formed by scattered waves are fundamental to the way in which they interact with their environment. For some waves, visualising this spatial distribution is straightforward. Surface waves on water are an inescapable part of the coastal landscape. The mode structures of sound waves in Chladni plates^1^, visualised by sand, are an accessible and popular physics demonstration. Direct measurement of the mode waveform is, however, not always so easy. Often spectral analysis of the scattering is more accessible, but the spectral information is not sufficient to understand the form of the modes in resonators and cavities. The question “Can one hear the shape of a drum?”^2^ has been negated^3,4^, meaning that the spectrum does not necessarily reveal the shape of the resonator, nor does it identify the spatial pattern of the mode. Still, the field distribution is of both great scientific interest and technological relevance. For example, maximising the coupling between a superconducting qubit and a mode in a cavity is dependent upon the qubit being at an antinode of the field^5^. In resonant enhanced nonlinear frequency mixing, the exact field distribution is required for efficient phase-matching between different interacting fields^6^.

For electromagnetic radiation, modes in open microwave cavities have long been probed by scanning a small perturbing element through the mode and measuring the change in its frequency^7^. Alternatively, direct measurement of the mode by an antenna has also been employed, allowing chaotic microwave systems to be explored^8,9^. However, these approaches require both physical access to the cavity, and an antenna or perturbing material smaller than the characteristic length scale of the mode structure. For dielectric optical resonators, these requirements appear at first sight to preclude the use of similar techniques.

The difficulties have been overcome in an elegant series of measurements by Wang et al.^10^. In their experiment, they study the optical mode distribution in a minuscule 20 μm dielectric resonator. Such resonators have been used for generating orbital angular momentum^11^ and chaos assisted phase-matching^12^, and have been shown to support both non-chaotic and chaotic modes. In this study a tightly focused pulsed blue (420 nm) laser beam perturbs the temperature and electron distribution in the dielectric and hence modifies the phase of the light travelling through this part. This small phase shift results in a dispersive shift of the mode which can be observed in the frequency domain. Crucially, this shift is proportional to the intensity of the mode field at the position of the blue laser spot. By scanning this probe across the resonator, the field distribution can be mapped (Fig. 1).

**Fig. 1:**
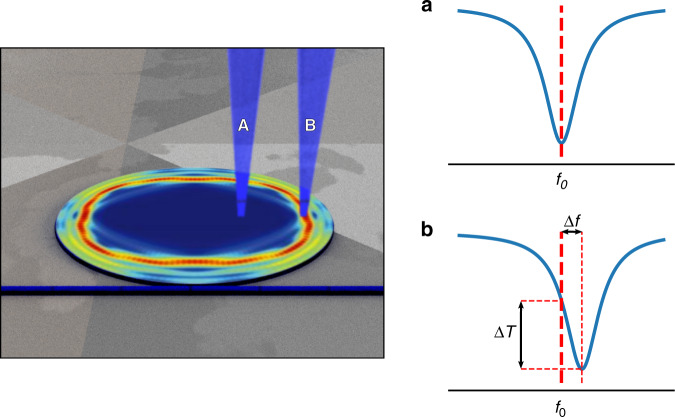
Experimental scheme. A chaotic mode in an oval microresonator is probed at its resonant frequency (left). When the microresonator is illuminated by a blue laser at a minimum of the mode, the resulting change in index has little effect upon the mode, and it remains at *f*_0_ (**a**). However, when the mode field is large at the illuminated spot, the shift Δ*f* is significant (**b**) and the transmission amplitude through the bus waveguide at *f*_0_ changes by Δ*T*

Employing this method, they first observe mode distributions in a circular microdisk, and by comparing their measurements to simulated whispering gallery modes, validate their technique and unambiguously identify the higher-order radial modes in the cavity. But the main focus of the study is deformed microresonators, which support both stable and chaotic modes^13^ and have a more complicated transmission spectrum. They identify four different mode families and with the help of the imaging realise that they belong to three modes localised on stable periodic orbits, one 4-reflection ‘diamond’ mode and two 6-reflection modes, as well as one, a so called ‘scar’ mode, localised on an unstable period orbit. They confirm these through numerical simulations and their Husimi projection.

In their final experiment they investigate chaos assisted tunnelling (CAT). This interesting phenomenon occurs when the field extent of a mode localised on a stable period orbit leaks into the surrounding ‘chaotic sea’. From the transport in the chaotic sea tunnelling into other stable period orbits is possible^14^. To test this idea an additional waveguide, port 3, is attached that does not overlap with the previously identified ‘diamond’ mode. When the ‘diamond’ mode is excited emission from port 3 provides direct experimental confirmation of CAT.

Chaotic transport leads on average to phenomena that do not obey time reversal^15^. As such the authors set out to study for the first time the time-reversal properties of CAT processes. Within the experimental limitations of their experiment, they could not identify the predicted broken time-reversal of the CAT process, as port 3 only probes a tiny subset of available states.

Through their visualisation method, the authors shed light on the exact electromagnetic field position in dielectric microcavities. In nonlinear processes, it is this field distribution that determines the ultimate efficiency of the process, and knowledge of this will help to engineer phase-matching for processes such as mmWave generation^6,16^ and microcomb generation^17^.
