# Author Correction: Visualising the heart of chaos

**DOI:** 10.1038/s41377-021-00672-w

**Published:** 2021-11-08

**Authors:** Nicholas J. Lambert, Harald G. L. Schwefel

**Affiliations:** 1 Department of Physics, University of Otago, Dunedin, New Zealand; 2 The Dodd-Walls Centre for Photonic and Quantum Technologies, Dunedin, New Zealand

**Keywords:** Microresonators, Scanning probe microscopy

Correction to: *Light: Science & Applications*

10.1038/s41377-021-00656-w published online 14 October 2021

Following publication of the article^1^, it is reported that this article contains some errors. The correction details are listed below.

1. Reference 10 has been updated as below.

10. Wang, S. et al. Direct observation of chaotic resonances in optical microcavities. *Light Sci. Appl.* **10**, 135 (2021).

2. Reference 16 has been updated as below.

16. Wang, B. C. et al. Towards high-power, high-coherence, integrated photonic mmWave platform with microcavity solitons. *Light Sci. Appl*. **10**, 4 (2021).

3. Reference 17 has been added to the reference list.

17. Chen, H. J. et al. Chaos-assisted two-octave-spanning microcombs. *Nat. Commun*. **11**, 2336 (2020).

4. The citations have been updated in the last sentence as below.

In nonlinear processes, it is this field distribution that determines the ultimate efficiency of the process, and knowledge of this will help to engineer phase-matching for processes such as mm Wave generation^6,16^ and microcomb generation^17^.

The original article has been updated.
